# Supplementary material for: Prevalence, severity, and risk factors of disability among adults living with HIV accessing routine outpatient HIV care in London, United Kingdom (UK): A cross-sectional self-report study
Source: PLoS One. 2022 May 12;17(5):e0267271. doi: 10.1371/journal.pone.0267271 (PMC9098035; doi:10.1371/journal.pone.0267271)
Supplement: S1 File — (PDF) [file pone.0267271.s001.pdf]

Date & Time	Study Code
	_ _ _ _

"DisHIV"
IRAS no: 236835

**Age**<br>(*years*)		
**Gender Identity**<br>(*please tick*)	Woman/Girl<br>Man/Boy<br>Transwoman/Transgirl<br>Transman/Transboy<br>Non-binary/Genderqueer/Agender/Gender fluid<br>Don't know<br>Prefer not to say<br>Other	☐<br>☐<br>☐<br>☐<br>☐<br>☐<br>☐<br>☐
**Sexual Orientation**<br>(*please tick*)	Bisexual<br>Gay/Lesbian<br>heterosexual/Straight<br>Don't know<br>Prefer not to say<br>Other	☐<br>☐<br>☐<br>☐<br>☐<br>☐
**Ethnicity**<br>(*see ethnicity codes page 4*)		
**Year of Diagnosis**<br>(*eg: 19XX format*)	Diagnosed late?<br>Please tick	☐
**Antiretroviral Therapy**<br>(*please circle*)	YES / NO	
**Undetectable Viral Load** (*please circle*)	YES / NO	
**Employment**<br>(*please tick*)	Self employed<br>Full time employed<br>Part time employed<br>On a government training scheme<br>Not working; available to start work in 2 weeks<br>Not working; looked for work in past 4 weeks<br>Waiting to start a new job<br>Unemployed<br>Retired<br>Maternity leave<br>Looking after family or home<br>Full time student/at school<br>Long term sick or disabled	☐<br>☐<br>☐<br>☐<br>☐<br>☐<br>☐<br>☐<br>☐<br>☐<br>☐<br>☐<br>☐

**Housing**<br>(*please tick*)	Owner occupied<br>Privately rented accommodation<br>Social rented housing<br>No fixed abode<br>Other …………………………………………..	☐<br>☐<br>☐<br>☐<br>☐
	Lives alone<br>Lives with friends<br>Lives with family<br>Lives with children<br>Other …………………………………………..	☐<br>☐<br>☐<br>☐<br>☐
Do you have adaptations at home to help with performing day-to-day activities?<br><br>YES/NO<br>Please Describe:		
**Education**<br>(*answer all 3 questions with circle or tick*)	Do you have any educational qualifications providing a certificate?<br>YES/NO<br><br>Do you have professional/vocational or other work related qualifications providing a certificate?<br><br>YES/NO<br><br>Was your highest qualification?<br>Degree level of above<br>Any other kind of qualification	 <br><br><br><br><br><br><br>☐<br>☐
**Transport**<br>(*please tick all that apply*)	How did you travel to your appointment today?<br>Car<br>Bus<br>Train<br>Tube<br>Bike<br>Walk<br>Other …………………………………………..	 ☐<br>☐<br>☐<br>☐<br>☐<br>☐<br>☐

**Health**<br>(*please tick*)	How would you rate your health today?<br>Poor<br>Fair<br>Good<br>Very Good	 ☐<br>☐<br>☐<br>☐
	Do you have any physical or mental health conditions or illnesses lasting or expected to last 12 months or more?<br>Yes<br>No	 ☐<br>☐
	Do any of your conditions or illnesses reduce your ability to carryout day-to-day activities<br>Yes, a lot<br>Yes, a little<br>Not at all	 ☐<br>☐<br>☐
	What is the total number of health conditions or illnesses you live with? (*place number in box*)	

**Care and Support**<br>(*please tick*)	I receive care from social services<br>I receive informal/unpaid care<br>I provide care for others (eg: family or friend)<br>I do not receive or provide care<br><br>Looking at page 5, are you at present receiving any of these state benefits in your own right, that is where you are the named recipient;<br><br>Working Age Benefits<br>Disability Benefits<br>Pensioner Benefits<br>Child Benefits<br>Social Fund<br>Other Benefits<br>One or more of these but I don't know which<br>Awaiting the outcome of a claim<br>None of these	☐<br>☐<br>☐<br>☐<br><br><br><br><br><br>☐<br>☐<br>☐<br>☐<br>☐<br>☐<br>☐<br>☐<br>☐
**Rehabilitation Services**<br>(*please read*)<br><br>"Rehabilitation services include any services or providers that address impairments (problems with body function or structure), activity limitations (problems carrying out a task), and social participation restrictions (problems interacting in a life situation)"	Have you received care from the following rehabilitation professionals in the last year?<br><br>Physiotherapy<br>Occupational Therapy<br>Speech and language therapy<br>Complimentary and alternative services (eg: acupuncture, Reiki)	 <br><br>☐<br>☐<br>☐<br>☐
**End of questions**		

What is your ethnic group?
Choose one option that best describes your ethnic group or background

White
1. English / Welsh / Scottish / Northern Irish / British
2. Irish
3. Gypsy or Irish Traveller
4. Any other White background, please describe

Mixed / Multiple ethnic groups
5. White and Black Caribbean
6. White and Black African
7. White and Asian
8. Any other Mixed / Multiple ethnic background, please describe

Asian / Asian British
9. Indian
10. Pakistani
11. Bangladeshi
12. Chinese
13. Any other Asian background, please describe

Black / African / Caribbean / Black British
14. African
15. Caribbean
16. Any other Black / African / Caribbean background, please describe

Other ethnic group
17. Arab
18. Any other ethnic group, please describe

Are you at present receiving any of these state benefits in your own right: that is, where you are the named recipient?

Working Age Benefits
Universal Credit
Housing Benefit
Working Tax Credit
Child Tax Credit
Income Support
Jobseekers Allowance
Employment and Support Allowance
Carer's Allowance

Disability Benefits
Personal independence Payment (including car allowance known as Motability)
Disability Living Allowance
Attendance Allowance
Severe Disablement Allowance
Incapacity Benefit
Industrial Injuries Disablement Benefit

Pensioner Benefits
Pension Credit
State Retirement Pension
Widow's Pension, Bereavement Allowance or Widowed Parrent's Allowance
Armed Forces Compensation Scheme
War Widow's/Widower's Pension

Child Benefits
Child Benefit
Guardian's Allowance
Maternity Allowance

Social Fund
A grant from the social fund for funeral expenses
A grant from the social fund for maternity expenses/Sure Start Maternity Grant
A budgeting loan or advance from DWP
A loan or grant from your local authority

Other Benefits
"Extended Payment" of Housing Benefit / rent rebate (4 week payment only)
Bereavement payment
Any National Insurance or State Benefit not mentioned earlier

This Section is for research use only
Participants should not complete

WHODAS 2.0				
**Simple Score**				**Complex Score**
**S1**		**S7**		**/100**
**S2**		**S8**		
**S3**		**S9**		
**S4**		**S10**		
**S5**		**S11**		
**S6**		**S12**		

HDQ			
**Physical**<br>Presence<br>Severity<br>Episodic	 /100<br>/100<br>/100	**Uncertainty**<br>Presence<br>Severity<br>Episodic	 /100<br>/100<br>/100
**Cognitive**<br>Presence<br>Severity<br>Episodic	 /100<br>/100<br>/100	**Day-to-Day Activities**<br>Presence<br>Severity<br>Episodic	 /100<br>/100<br>/100
**Mental & Emotional**<br>Presence<br>Severity<br>Episodic	 /100<br>/100<br>/100	**Social Inclusion**<br>Presence<br>Severity<br>Episodic	 /100<br>/100<br>/100
**Disability Presence Score**		**/100**	
**Disability Severity Score**		**/100**	
**Disability Episodic Score**		**/100**	
**Total HDQ Score**		**/100**	
