# Supplementary material for: Prevalence, severity, and risk factors of disability among adults living with HIV accessing routine outpatient HIV care in London, United Kingdom (UK): A cross-sectional self-report study
Source: PLoS One. 2022 May 12;17(5):e0267271. doi: 10.1371/journal.pone.0267271 (PMC9098035; doi:10.1371/journal.pone.0267271)
Supplement: Attachment — Submitted filename: Response_to_Reviewers.docx [file pone.0267271.s003.docx]

PONE-D-21-28696

Prevalence, severity, and risk factors of disability among adults living with HIV accessing routine outpatient HIV care in London, United Kingdom (UK): A cross-sectional self-report study.

Thank you to the journal and peer reviewers for your positive and constructive feedback. The authors are grateful for the time taken to review this manuscript and have commented on your feedback below in BLUE.

**Journal Requirements:**

1. Please ensure that your manuscript meets PLOS ONE's style requirements, including those for file naming.

RESPONSE: Thank you. We have ensured the manuscript meets PLOS ONE’s style requirements. We have amended the title, authors, and affiliations page, amended abstract headings, amended table names and citations, introduced the supplemental file section within the manuscript, amended the supplemental file names and citations, and amended all file names.

2. Please include additional information regarding the survey or questionnaire used in the study and ensure that you have provided sufficient details that others could replicate the analyses. For instance, if you developed a questionnaire as part of this study and it is not under a copyright more restrictive than CC-BY, please include a copy, in both the original language and English, as Supporting Information.

RESPONSE: Thank you. We have included as a supplemental file (S1 File) the demographic and HIV characteristic questionnaire used in the study. This has been cited on line 333, and included in supplemental file section on line 1131.

3. Thank you for stating the following financial disclosure:

“This study/project is funded by the National Institute for Health Research (NIHR) Masters of Research (MRes) Programme. The views expressed are those of the author(s) and not necessarily those of the NIHR or the Department of Health and Social Care.”

Please state what role the funders took in the study.  If the funders had no role, please state: "The funders had no role in study design, data collection and analysis, decision to publish, or preparation of the manuscript."

If this statement is not correct you must amend it as needed.

Please include this amended Role of Funder statement in your cover letter; we will change the online submission form on your behalf.

RESPONSE: Thank you. We confirm that “The funders had no role in study design, data collection and analysis, decision to publish, or preparation of the manuscript.” We have updated the cover letter to include these statements. Thank you for changing the online submission form on our behalf.

4. In your Data Availability statement, you have not specified where the minimal data set underlying the results described in your manuscript can be found. PLOS defines a study's minimal data set as the underlying data used to reach the conclusions drawn in the manuscript and any additional data required to replicate the reported study findings in their entirety. All PLOS journals require that the minimal data set be made fully available. For more information about our data policy, please see <http://journals.plos.org/plosone/s/data-availability>.

Upon re-submitting your revised manuscript, please upload your study’s minimal underlying data set as either Supporting Information files or to a stable, public repository and include the relevant URLs, DOIs, or accession numbers within your revised cover letter. For a list of acceptable repositories, please see <http://journals.plos.org/plosone/s/data-availability#loc-recommended-repositories>. Any potentially identifying patient information must be fully anonymized.

Important: If there are ethical or legal restrictions to sharing your data publicly, please explain these restrictions in detail. Please see our guidelines for more information on what we consider unacceptable restrictions to publicly sharing data: <http://journals.plos.org/plosone/s/data-availability#loc-unacceptable-data-access-restrictions>. Note that it is not acceptable for the authors to be the sole named individuals responsible for ensuring data access.

We will update your Data Availability statement to reflect the information you provide in your cover letter.

RESPONSE: Thank you. We have uploaded our study’s minimal underlying data set to the public repository DRYAD. The data can be accessed on the URL and DOI below. We have updated the cover letter to include the links to the study’s minimal underlying data set.

<https://datadryad.org/stash/share/5o5ixIjJZbIjsqO9363pRnIPIg7LVUngZTYnEyX7JUc>

doi:10.5061/dryad.qnk98sfjn

5. Please review your reference list to ensure that it is complete and correct. If you have cited papers that have been retracted, please include the rationale for doing so in the manuscript text, or remove these references and replace them with relevant current references. Any changes to the reference list should be mentioned in the rebuttal letter that accompanies your revised manuscript. If you need to cite a retracted article, indicate the article’s retracted status in the References list and also include a citation and full reference for the retraction notice.

RESPONSE: Thank you. We have reviewed the reference list to ensure it is complete and correct. We have made the following modifications:

1) Reference 8 hyperlink no longer worked, so we have updated this citation with the correct information: “Yin Z, Kall M, Skingsley A, Dalpech V. Over half of people in HIV care in the United Kingdom by 2028 will be aged 50 years or above. P127. HIV Medicine. 2017;18:S1.”
2) Reference 46 “Washington Group on Disability Statistics. Washington Group questions and the Sustainable Development Goals. Dr Daneil Mont. 2017. Available: <http://www.washingtongroup-disability.com/washington-group-blog/sdgs/>” has been removed because the link is no longer accessible. This reference has been replaced with reference 41 (Madans, 2011) (Page 31, Line 780), as this reference provides the correct information required in relation to the Washington Group Short Set questions being recommended by the United Nations. All subsequent references have been updated according to correct numerical values following this change.
3) Reference previously numbered 71 (now 70) hyperlink not functioning. This has been updated to “Auzenbergs M, Delpech V, Gold D, Kall M, Petretti S, Smithson K, et al. Positive Voices, the national survey of people living with HIV; Changing Perceptions: "Talking about HIV and our needs". 2018. Available: <https://www.nat.org.uk/sites/default/files/publications/web_PV_Changing%20Perceptions-needs-report.pdf>
4) Reference previously number 76 (now 75) hyperlink not functioning, because the Government Statistical Service updated their website. Has been updated to “Government Statistical Service. Education attainment and qualifications. 2020. Available: <https://gss.civilservice.gov.uk/policy-store/educational-attainment/>”
5) We have included a new sentence in the discussion (line 758-9), to include new available evidence. The sentence is “women living with HIV also experience high levels of poverty, psychological distress, and social isolation” with a new citation [100]. All subsequent citations are numerically updated.

**Reviewers Comments**

Reviewer #1:

1. The study reported the prevalence and severity disability and risk factors among adults living with HIV in London, United Kingdom; in order to identify areas to target interventions, reduce disability, and optimise health and function. This study provides useful information and contributes substantially to the epidemiology of HCV in preparation of the elimination in the country. The manuscript is well prepared and informative with clear statement of objectives. Similarly, the methods section is appropriately explained and results section is prepared well with minor mistakes as indicated below. While publication is recommended, the manuscript requires minor revision.

RESPONSE: Thank you for the positive and constructive feedback. Our responses to the mistakes and minor revisions are below.

2. The study mentioned that participants were on ART longer than 6 months, however they did not assess whether specific ART is associated with disability.

RESPONSE: Thank you. We did not collect data on the types of medications (ART) participants were taking to treat HIV. The examination of different ART regimens, as potential risk factors for disability, was therefore not possible within this study. However, this is an important research consideration that could be included within future research into the disability experiences of people living with HIV.

3. Furthermore, although data on concurrent health conditions were collected and participants reporting living with a median of two concurrent health conditions in addition to HIV, additional data on the specific concurrent health condition would have added value to enhance the understanding of the risk factors associated with HIV and disability.

RESPONSE: Thank you. We collected data on the number of concurrent health conditions participants were living with in addition to HIV. However, we did not collect data on the types of concurrent health conditions people were living with. Therefore it was not possible to report on the types of concurrent conditions people were living with. We agree that this additional data would have added value, and will be considered in future research into the disability experiences of people living with HIV.

4. The abstract is too long and it does not conform to PLOS one standards

RESPONSE: Thank you. We have amended the abstract length and it now conforms with PLOS ONE standards.

5. Page 15: Table 1: Participant characteristics
Gender identity: singular vs plural eg Man vs Women

RESPONSE: Thank you. We have amended the table to ensure that participant characteristics are singular for both Man and Woman.

6. Page 18, the authors did not follow PLOS instructions guidelines for labelling supplementary files

RESPONSE: Thank you. We have amended the labelling of supplementary files to follow PLOS ONE instruction guidelines. The sentence has been updated to “The WHODAS simple scores in response to all 12-items are shown in S1 Table” (page 18, line 507). We have also included an additional supplementary file (S1 File) “Demographic and HIV questionnaire”, which is cited in the manuscript (page 10, line 333).

7. Page 18, the authors state that “Difficulty levels across all WHODAS items were “mild difficulty” n=333 (38.8%)…. while page 28 in the discussion they state “In our study, 50.5% reported 4 or more functional limitations with mostly mild (36.8%) or ……

RESPONSE: Thank you. We appreciate identification of this typo. We have updated the discussion (page 28, line 647) to say the correct value 38.8%.

8. There are no line numbers used in the document- the authors did not following PLOS instructions guidelines

RESPONSE: Thank you. We have amended the document to include line numbers, and follow PLOS ONE instruction guidelines.

Reviewer #2:

1. The paper is very important and timely for the improvement of care for people with living with HIV. Though it's more or less exploratory, I believe has pointed a light to a potentially serious problem.

RESPONSE: Thank you for the positive feedback.

2. The paper can improve with added attention to editing. Also, the data behind findings requirement is not sufficiently fulfilled.

RESPONSE: Thank you. We have amended the paper in response to the journal requirements and reviewer responses. We have uploaded our study’s minimal underlying data set to the public repository DRYAD. The data can be accessed on the URL and DOI below. We have updated the cover letter to include the links to the study’s minimal underlying data set.

<https://datadryad.org/stash/share/5o5ixIjJZbIjsqO9363pRnIPIg7LVUngZTYnEyX7JUc>

doi:10.5061/dryad.qnk98sfjn
